# Letter to the editor: critical evaluation of the reliability of DNA methylation probes on the illumina MethylationEPIC v1.0 BeadChip microarrays

**DOI:** 10.1080/15592294.2024.2411470

**Published:** 2024-10-04

**Authors:** Julian Hecker, Scott T. Weiss, Jessica A. Lasky-Su, Dawn L. DeMeo, Christoph Lange

**Affiliations:** a Channing Division of Network Medicine, Department of Medicine, Brigham and Women’s Hospital and Harvard Medical School, Boston, MA, USA; b Department of Biostatistics, Harvard T.H. Chan School of Public Health, Boston, MA, USA

**Keywords:** Batch effects, DNA methylation, slide effects, methylation array

In their recent work, Zhang et al. comprehensively evaluated the reliability of DNA methylation probes on the Illumina MethylationEPIC v1.0 BeadChip microarray using intraclass correlation coefficients (ICCs) calculated based on duplicated samples [1]. Using the observed ICCs, they classified probes into the four reliability categories ‘Excellent,’ ‘Good,’ ‘Fair,’ and ‘Poor,’ and shared their results with the readership, creating a valuable resource for future studies. Their downstream analyses demonstrated that higher probe reliability is associated with more consistent results in epigenome-wide association studies (EWAS) and other integrative omics studies. Importantly, Zhang et al. noted the non-ignorable impact of batch effects when analyzing DNA methylation microarray data and considered duplicates measured on different methylation plates. In line with this argument, they observed that the median ICC increased from 0.325 to 0.733 when considering duplicated samples placed on the same methylation plate.

Batch effects are complex and can be described at different levels, including plate, slide, or position [2]. Recently, we focused on batch effects defined on the (glass) slide level and aimed to quantify their contribution to probe-specific variation utilizing multiple DNA methylation array datasets, especially based on the EPIC v1.0 array [3]. We observed a consistent and systematic pattern of slide effects and partitioned CpGs into the five categories $S_{0-20},$ $S_{20-40},$ $S_{40-60},$ $S_{60-80},$ and $S_{80-100},$ representing an increasing impact of slide effects (without modelling other batch effects simultaneously). We proposed to utilize this information to improve slide effect adjustment approaches. For example, performing adjustment approaches such as ComBat [4,5] for these sets separately, or applying singular value decomposition/principal component analysis (PCA) directly or, with increased weight, to CpGs highly impacted by slide effects (see the R package *SeffCovar*).

Zhang et al. provided the ICC values and category information for 640,960 probes. By merging their results with ours, we obtained a combined mapping for a total of 639,171 probes (>99.7% overlapping). Among these probes, 349,472 were labelled with ‘Poor’ reliability by Zhang et al. Interestingly, 143,813 (~41%) of these probes lie in the sets $S_{40-60},$ $S_{60-80},$ or $S_{80-100}$, indicating, based on our results, that slide effects can explain a substantial fraction of their variation. In addition, the proportion of CpGs with poor reliability increases with increasing strength of slide effects across the sets $S_{0-20},$ $S_{20-40},$ $S_{40-60},$ $S_{60-80},$ and $S_{80-100}$. We visualized this in Figure 1. Figure 1 also contains the analogous visualization of probe ICC values provided by Bose et al. [6], Higgins-Chen et al. [7], Sugden et al. [8], and Logue et al. [9], spanning the EPIC and 450K array. The ICC results for the analyses by Sugden et al. and Logue et al. were obtained from the supplemental material of Higgins-Chen et al. [7]. We utilized the ICC values provided by the respective studies and labelled the same reliability categories based on ICC value cutoffs as in Zhang et al. (while noting and neglecting that Zhang et al. considered uncertainty-adjusted ICC values, whereas not all other studies used this additional adjustment). Overall, the observations indicate that not all but a substantial fraction of the CpGs labelled with poor reliability is strongly impacted by slide effects.

**Figure 1.**
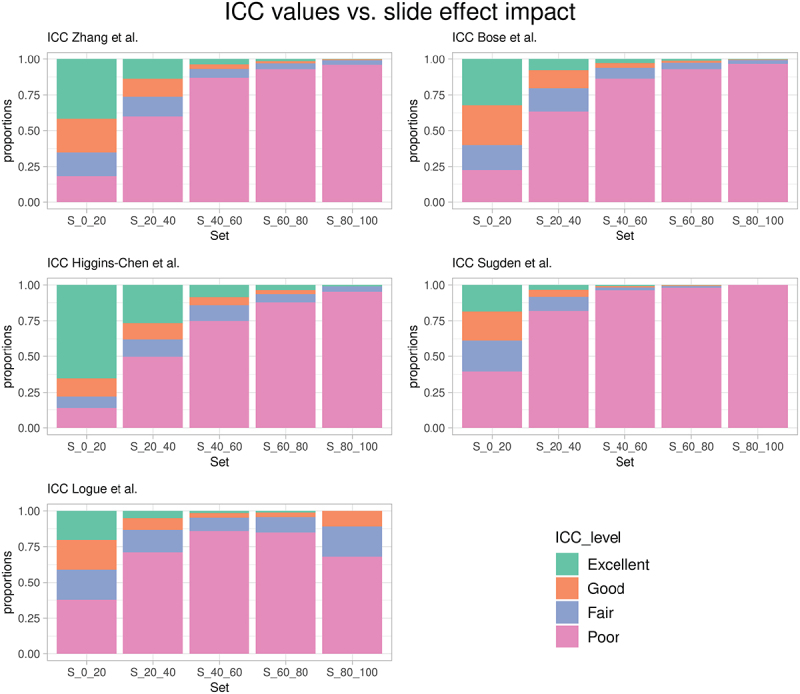
Bar plots showing the proportions of the CpGs in the reliability categories ‘Excellent,’ ‘Good,’ ‘Fair,’ and ‘Poor’ [1], for different studies [1,6–9] and stratified by the sets S_0–20_, S_20–40_, S_40–60_, S_60–80_, and S_80–100_ [3].

Batch effects can produce false positive findings if the batch structure is aligned with experimental comparisons. On the other hand, they can also significantly reduce statistical power due to the increased technical variation in the data. The comparisons visualized in Figure 1 suggest that a significant fraction of CpGs with low reliability (measured using ICCs) is highly impacted by slide effects. Therefore, an important question for future studies is to what extent the reliability of specific CpGs depends on prior and sufficient adjustment for batch effects on different levels, including a potentially critical role of slide effects. Related, Bose et al. compared ICC values before and after adjusting for chip effects [6], but the relationship between the impact of different batch effects and the ICC measure is hard to predict and depends on the composition of the overall probe-specific variation. We suggest that improved adjustment for batch effects on different levels is needed and might increase the likelihood of unravelling biologically relevant associations with CpGs disproportionally affected by more complex batch effects.

## Data Availability

The ICC values and the CpG sets were obtained from the supplementary material of the respective publications by Zhang et al. [1], Hecker et al. [3], Bose et al. [6], and Higgins-Chen et al. [7], as well as from the SeffCovar R package (https://github.com/julianhecker/SeffCovar).
